# Retractions of publications in radiomics: An underestimated problem?

**DOI:** 10.1007/s00330-025-12231-7

**Published:** 2025-12-20

**Authors:** Aydin Demircioğlu

**Affiliations:** Institute of Diagnostic and Interventional Radiology and Neuroradiology, University Hospital Essen, Essen, Germany

**Keywords:** Radiomics, Research Integrity, Methodology, Radiology, Machine Learning

## Abstract

**Abstract:**

Radiomics is increasingly explored as a tool for improving diagnosis, prognosis, and treatment planning. However, concerns exist about the reproducibility and methodological rigor of its studies. The integration of high-dimensional radiomic features and machine learning makes the field prone to unintentional errors that may warrant retraction. Despite a rising number of retractions in science overall, no dedicated study has examined retractions specifically within radiomics. Therefore, this study aimed to review retracted radiomics publications and identify the characteristics and reasons for their retraction. We systematically searched six databases (Crossref, Retraction Watch Database, OpenAlex, PubMed, Scopus, Web of Science) and identified 93 retracted radiomics publications, of which 20 were included. These articles were analyzed with respect to publisher, country of origin, dates, citation counts, and reasons for retraction. Retraction rates were then estimated and compared with those in general radiology. Our findings indicate that a disproportionate number of retractions are linked to specific publishers and countries (particularly China and India), with overall low citation counts (median 4.0 citations). Retractions peaked sharply in 2023, followed by a strong decline. Many retraction notes lack a clear explanation for the retraction. Estimated retraction rates in radiomics were lower than in general radiology (6.7 vs 7.4 per 10,000 publications). Notably, no major radiological or oncological journal appears to have retracted a radiomics publication. Given that radiomics demands higher, interdisciplinary expertise, this suggests a gap, implying that flawed research may yet have to be retracted.

**Key Points:**

***Question*** *Considering the technical complexity of radiomics studies and their susceptibility to unintentional errors, how do their retraction rates compare to those in general radiology*?

***Findings*** *Retractions in radiomics were disproportionately linked to specific publishers and countries; however, no retractions appeared in major journals. Estimated retraction rates were lower than those for general radiology publications*.

***Clinical relevance*** *A potential gap in the number of retracted radiomics studies was identified, implying that flawed research in the field may not yet have been addressed*.

**Graphical Abstract:**

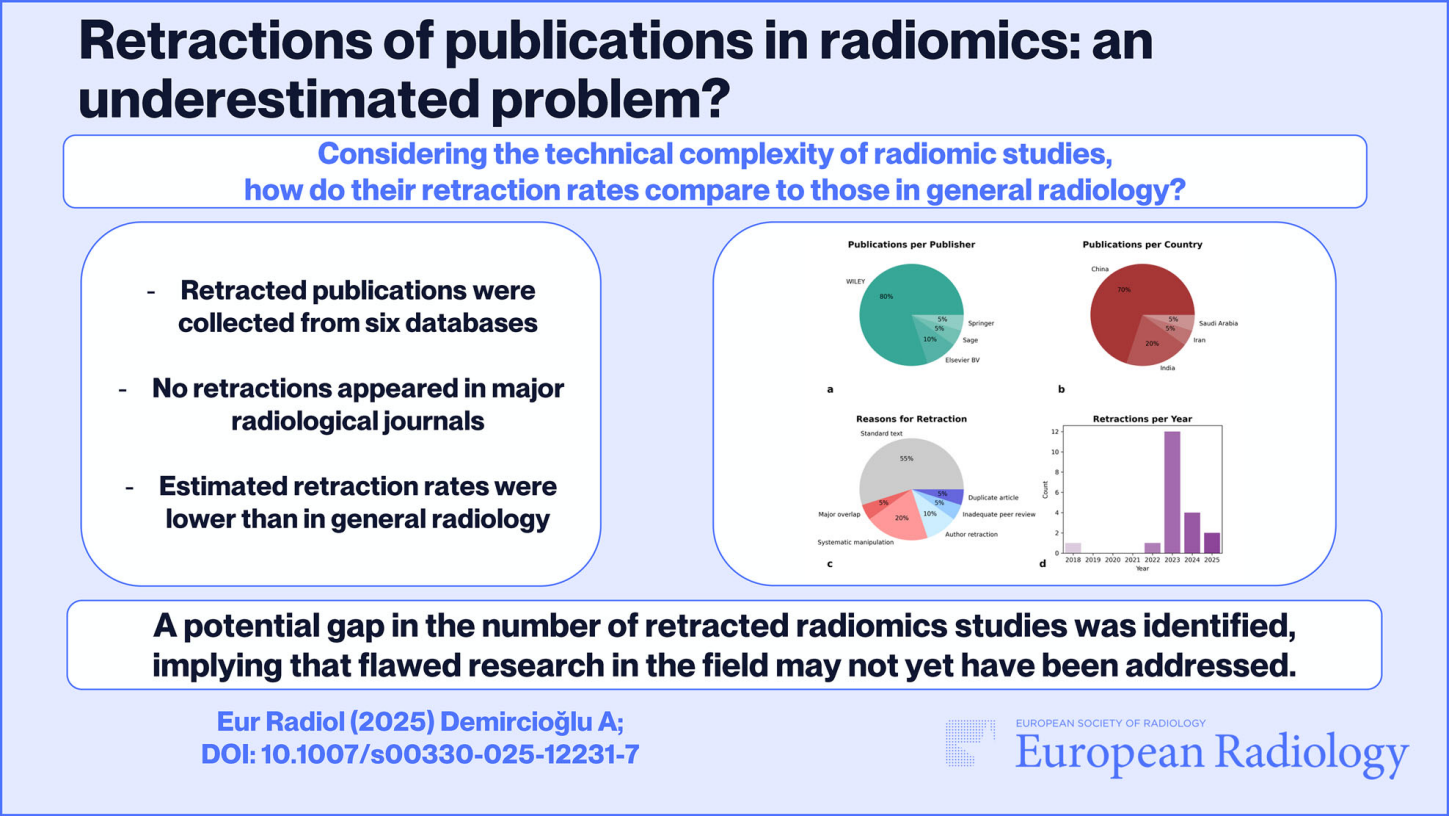

## Introduction

Over the years, radiomics has been applied to a wide range of tasks, including diagnosis, prognosis, and related objectives [1, 2]. Conceptually, radiomics can be understood as a quantitative image analysis technique that extracts high-dimensional features from radiological imaging data, originating in the 1970s [3]. Its central premise is to extract quantitative features from imaging data, identify those that may serve as biomarkers, and develop predictive models for clinical outcomes of interest.

One of the major challenges in radiomics is its reproducibility, that is, the extent to which results vary significantly depending on factors such as imaging acquisition parameters, scanner models, image reconstruction algorithms, and methodology [4]. If not adequately controlled, these factors can lead to inconsistent findings, thereby limiting the generalizability and clinical applicability of radiomics models [5, 6]. In particular, the absence of standardized pipelines for feature extraction, preprocessing, and model evaluation further complicates model development and can introduce methodological flaws, such as data leakage, which can severely compromise a model’s validity and result in false-positive findings. Indeed, recent studies suggest that these issues may be widespread, potentially affecting 10–15% of all radiomics publications [7–10]. This concern is further exacerbated by the lack of data and code sharing in many studies, which makes it difficult, if not outright impossible, to verify the validity of the methods used and the results reported [11]. Moreover, many radiomics models lack rigorous validation on independent or external datasets, which hinders the assessment of their generalizability and, consequently, their translation into clinical routine.

Given these challenges, it is plausible to expect that a portion of published radiomics studies may contain errors or invalid results. According to the guidelines of the International Committee on Publication Ethics (COPE), such publications should be retracted when there is “clear evidence that the findings are unreliable, either as a result of major error (e.g., miscalculation or experimental error), or as a result of fabrication (e.g., of data) or falsification (e.g., image manipulation)” [12].

Recent research has shown that retraction rates in science, particularly in the medical field, are increasing [13, 14]. This study was conducted to assess how many radiomics publications have been retracted and to identify the reasons for their retraction.

## Materials and methods

### Databases

Six databases were used to identify retracted publications [15]: The Retraction Watch Database (RWD), Crossref, OpenAlex, PubMed, Scopus, and Web of Science (WoS). All searches were performed by the corresponding author on 21st October 2025. All data and code are available in a GitHub repository at https://github.com/aydindemircioglu/radReproducible.

### RWD

The database of the RWD was downloaded and filtered using the keyword ‘radiomics’ (case-insensitive).

### Crossref

The Crossref database was searched using the query term “radiomics” via its application programming interface (API) and by applying the filter “update-type:retraction”.

### OpenAlex

The OpenAlex database was queried using the term “radiomics” in the “Title&Abstract” field. Retracted publications were identified using the “retracted” filter.

### PubMed

The PubMed database was queried with the search term “radiomics”, and the results were then filtered by the article type “Retracted publication”.

### Scopus

The Scopus database was queried using the term “(radiomics)” in the ALL field, after which the search was limited to the article type “Retracted”.

### WoS

An advanced search was performed using the query “Radiomics”, followed by filtering the article type to “Retracted publication”.

### Data processing

After collecting all results, the data were merged using a Python script. The articles were then screened to determine whether they constituted original research applying standard radiomic techniques. The criterion was the extraction of a large number of radiomics features. For example, a paper measuring nodule sizes in lung cancer patients was not considered radiomics. This criterion also excluded studies that used the term radiomics but did not extract any radiomic features (as defined by the Image Biomarker Standardization Initiative [16]) and instead applied deep learning methods directly to the imaging data.

### Characteristics of retracted papers

After identifying the relevant publications, the following information was collected: publisher, country of origin based on the first author, the dates of publication and retraction, number of citations (further divided into before and after retraction), and the reason for the retraction as stated in the corresponding retraction note.

Citation data were retrieved using Google Scholar, which, unlike other sources, includes non-peer-reviewed materials, providing a more comprehensive measure of an article’s impact. The division between citations before and after retraction was primarily based on the year of the publication, as a more detailed timeline is often unavailable, for example, when articles are published ahead of print.

The reasons for retraction were determined based on the corresponding retraction notes and categorized into three groups: First, misconduct by the authors, such as fraudulent publication. Second, errors by the publisher, including an inadequate review process. Third, cases where the reason was unclear, as the retraction note did not explicitly state who was responsible but included standard phrases like ‘inadequate citing’ or ‘article manipulation’, making it impossible to determine whether the misconduct was intentional.

### Retraction rates

Retraction rates for radiomic publications were calculated by dividing the number of retracted publications by the total number of radiomic publications. Because the Scopus dataset included many false positive publications, which would bias the estimation, the search was repeated with the term ‘radiomics’ restricted to the title, abstract, and keywords.

Retraction rates for the radiology field over the same time period (since 2012) were determined similarly. For this, analogous searches were conducted as follows: in Crossref, the search term “Radiology” was used in combination with a filter to select only the publications after 2012. In OpenAlex, “Radiology OR Medical Imaging” was entered in the “Title&Abstract” field, and the search was narrowed down to publications after 2012. In PubMed, the search term ‘(“Radiology”[Mesh] OR radiolog*[Title/Abstract] OR “Medical Imaging”[Title/Abstract]) AND (“2012”[Date—Publication] : “2026”[Date - Publication])’ was used to identify the total number of radiology publications, which was then narrowed to include only retracted publications. Similarly, Scopus was queried with ‘TITLE-ABS-KEY(radiolog* OR “medical imaging”) AND PUBYEAR > 2011 AND PUBYEAR < 2026’, and WoS with ‘TS = (radiolog* OR “medical imaging”) AND PY = (2012–2026)’. The RWD dataset was excluded as it contains only retracted publications.

Since radiomics publications are likely included within the broader category of radiology publications, the numbers were corrected by subtracting the radiomics counts from the radiology counts, followed by recomputation of the retraction rates.

### Evaluation

The data were then summarized graphically, including publisher, country of origin, reason for retraction, retractions per year, and citation counts. Additionally, time-to-retraction was calculated and plotted.

## Results

Overall, 93 retracted publications were identified, of which 20 met the inclusion criteria and were included in this study (Fig. 1 and Table 1).

**Fig. 1:**
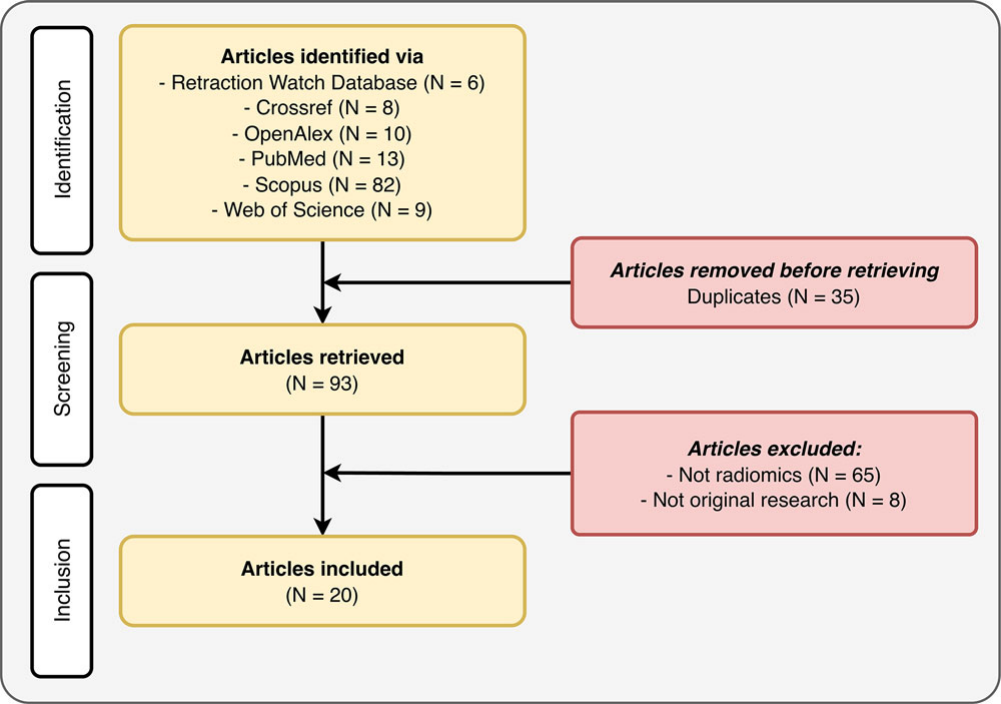
Flowchart of the review

**Table 1:** Retracted publications

Authors	Publication year (retraction year)	Journal	Publisher	Country	Given the reason for withdrawal	Total citations (before/after retraction)	DOI
Abdollahi et al	2018 (2018)	Medical Physics	Wiley	Iran	Major overlap	1 (0/1)	10.1002/mp.13166
Chen et al	2021 (2022)	Medical Physics	Wiley	China	Duplicate article	3 (1/2)	10.1002/mp.15035
Das et al	2021 (2025)	Concurrency and Computation: Practice and Experience	Wiley	India	Standard text	51 (43/8)	10.1002/cpe.6501
Khadidos et al	2021 (2023)	Applied Bionics and Biomechanics	Wiley	Saudi Arabia	Systematic manipulation	24 (23/1)	10.1155/2021/4520450
Sheng et al	2021 (2023)	Journal of Healthcare Engineering	Wiley	China	Standard text	2 (2/0)	10.1155/2021/6088322
Xu et al	2021 (2023)	Stem Cells International	Wiley	China	Standard text	14 (8/6)	10.1155/2021/2263469
Chen et al	2022 (2023)	Contrast Media & Molecular Imaging	Wiley	China	Standard text	8 (3/5)	10.1155/2022/7642511
He et al	2022 (2023)	Oxidative Medicine and Cellular Longevity	Wiley	China	Standard text	4 (3/1)	10.1155/2022/7261786
Jing et al	2022 (2023)	BioMed Research International	Wiley	China	Systematic manipulation	5 (5/0)	10.1155/2022/4667117
Pareek et al	2022 (2023)	Advances in Materials Science and Engineering	Wiley	India	Systematic manipulation	27 (13/14)	10.1155/2022/1008652
Sheng et al	2022 (2023)	Scanning	Wiley	China	Systematic manipulation	6 (5/1)	10.1155/2022/5314225
Wang et al	2022 (2023)	Computational and Mathematical Methods in Medicine	Wiley	China	Standard text	4 (2/2)	10.1155/2022/2155132
Xie et al	2022 (2023)	Journal of Healthcare Engineering	Wiley	China	Standard text	3 (3/0)	10.1155/2022/3107965
Yang et al	2022 (2023)	Contrast Media & Molecular Imaging	Wiley	China	Standard text	2 (1/1)	10.1155/2022/3417480
Anand et al	2023 (2024)	BioMed Research International	Wiley	India	Standard text	19 (5/14)	10.1155/2023/3913351
Liang et al	2023 (2024)	Functional and Integrative Genomics	Springer	China	Standard text	2 (2/0)	10.1007/s10142-023-01011-5
Prusty et al	2023 (2024)	Journal of Intelligent and Fuzzy Systems	Sage	India	Inadequate peer review	7 (5/2)	10.3233/JIFS-223265
Xiu et al	2023 (2023)	Journal of Oncology	Wiley	China	Standard text	0 (0/0)	10.1155/2023/8607062
Liu et al	2024 (2024)	Neuroscience Informatics	Elsevier	China	Author retraction	0 (0/0)	10.1016/j.neuri.2024.100163
Li et al	2025 (2025)	Cancer Treatment and Research Communications	Elsevier	China	Author retraction	1 (0/1)	10.1016/j.neuri.2024.100163

### Characteristics of retracted papers

The majority of retracted radiomic publications were published in journals by Wiley (*N* = 16, 80%) (Fig. 2a). Similarly, most retracted publications originated from two countries (Fig. 2b): China (*N* = 14, 70%), and India (*N* = 4, 20%). Two publications originated from other countries (*N* = 2, 10%).

**Fig. 2:**
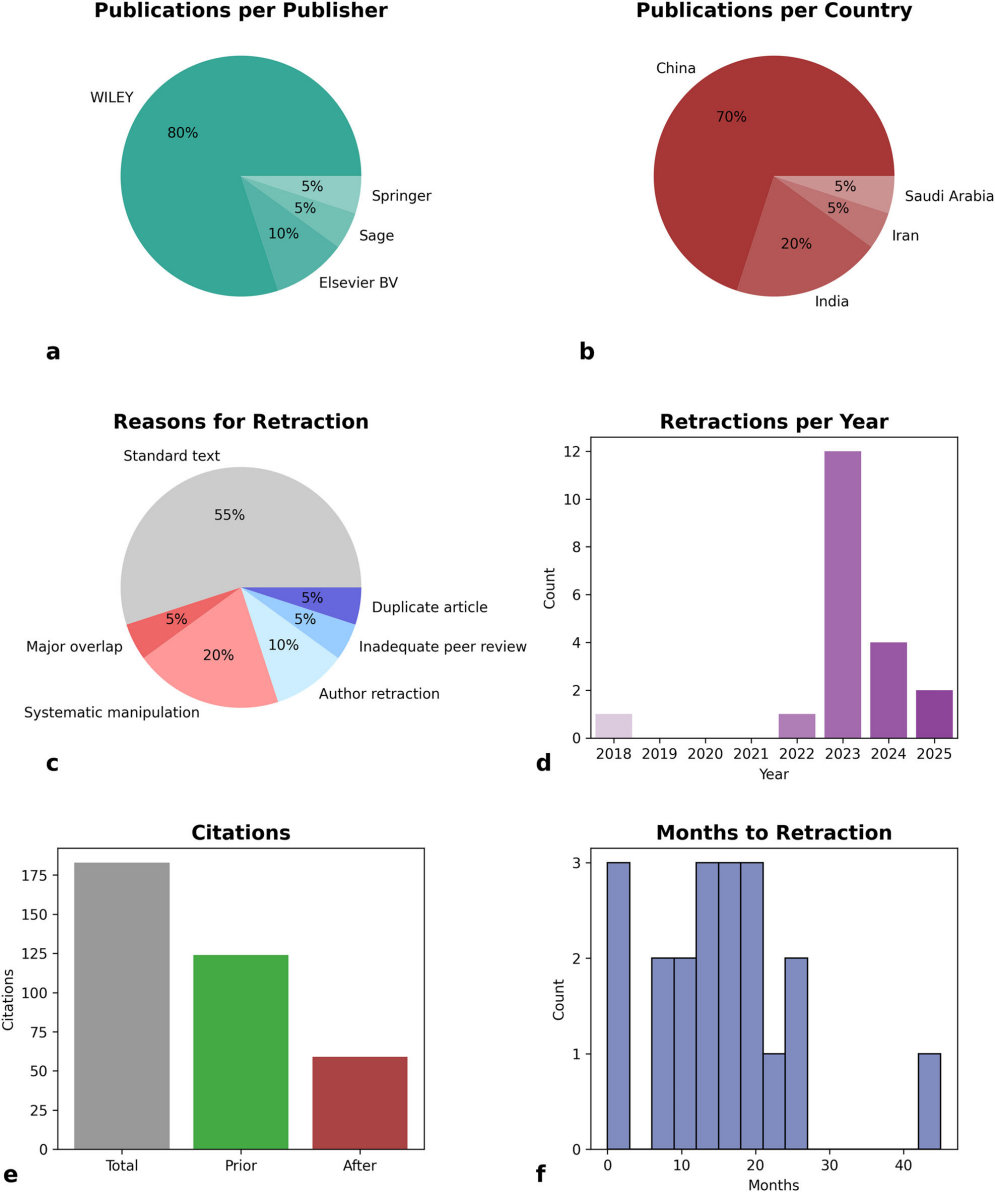
Characteristics of retracted papers. **a** Publications per publisher, (**b**) publications per country, (**c**) reasons for retraction, (**d**) retractions per year, (**e**) citations (total, before, after), and (**f**) retractions per month. In panel **c**, the reasons for retraction were color-coded: red indicated retractions due to clear misconduct by the authors, blue indicated errors attributable to the publisher, and gray was used for cases where a standard text was provided without a clear indication of whether the error was due to the authors, editors, or publisher

In most cases, the reasons for retraction were unclear (Fig. 2c). In 11 publications (55%), a standard text was used that did not allow for clear identification of whether misconduct or unintentional error had occurred, nor did it clarify whether the responsibility lay with the authors or with the review process, reviewers, or editors. In five publications (20%), the retraction notes explicitly charged the authors with fraudulent behavior. In two publications (10%), the reasons were on the part of the publisher, while in another two publications (10%), the authors (or the editor) retracted the publication for partly unclear reasons.

Most retractions occurred in 2023 (*N* = 12, 60%), with many taking place shortly before and after (Fig. 2d). The first publication was retracted only in 2018 (5%). Citation counts were generally low (median: 4.0 citations), with the highest citation count being 51 (Fig. 2e, Table 1). Citations for nearly all publications dropped by nearly half following retraction, including the two articles retracted due to publisher errors. Time to retraction ranged from one week to 43 months, with a median of 14.5 months (Fig. 2f).

### Retraction rates

Retraction rates for radiomic publications varied by database, ranging between 2.6 and 15.2 per 10,000 publications (mean: 6.7; Table 2). In comparison, rates for radiological papers were generally higher, except in the Scopus (uncorrected) and Crossref databases, and ranged from 3.2 to 10.0 (mean: 7.4). Due to the relatively low number of radiomics publications, retraction rates for non-radiomic radiology publications did not change substantially (range 3.0–10.5, mean: 7.4).

**Table 2:** Retraction rates

Database	Total radiomic papers	Retracted radiomic papers	Radiomic retraction rate [per 10,000]	Total radiological papers	Retracted radiological papers	Radiological retraction rate [per 10,000]	Total non-radiomic radiological papers	Retracted non-radiomic radiological papers	Non-radiomic radiological retraction rate [per 10,000]
Crossref (term: radiology)	14,475	8	5.5	182,604	59	3.2	168,129	51	3.0
OpenAlex	24,460	10	4.1	352,300	212	6.0	327,840	202	6.2
PubMed	16,529	13	7.9	226,636	198	8.7	210,107	185	8.8
Scopus (uncorrected)	54,088	82	15.2	404,923	345	8.5	350,835	263	7.5
Scopus (corrected)	19,430	5	2.6	423,751	347	8.2	404,321	342	8.5
WoS	18,138	9	5.0	220,934	221	10.0	202,796	212	10.5

Comparison of the retraction rates of radiomic and radiological publications. The number of radiomic papers represents all publications without any screening to allow for an unbiased comparison. The non-radiomic radiological retraction rates were calculated by excluding the radiomics papers from the total radiological publications. Because Crossref does not support Boolean operators, only the search term “Radiology” was used. As the Scopus results included a large number of false positives, which could positively bias the retraction rates, a second search was performed using the TITLE-ABS-KEY field instead. All retraction rates are reported as the number of retracted papers per 10,000 publications

## Discussion

In this study, retracted publications in radiomics collected from six databases were reviewed. While the overall retraction rate was low, a disproportionate number of retractions were associated with specific publishers and countries. Transparent explanations for the retractions were lacking in nearly all retraction notes.

Nearly all retracted articles were published in journals by Wiley around the year 2023, during which many fraudulent papers across all fields were retracted within a short time period [17]. Notably, most retracted radiomic papers appeared in non-radiological or non-oncological journals. Not a single radiomics publication was retracted by any major radiological or oncological journals where most radiomics research is published. Furthermore, the majority of retracted publications originated from China or India. While the total number of retractions remains small and the reasons are unclear, this pattern could suggest a more systematic issue [18]. As expected, most retracted papers received few citations, and, as observed by Furman et al, citations do not cease immediately after retraction but gradually decrease [19].

Estimated retraction rates for radiomics publications varied more than those for radiology, likely due to fewer retractions in radiomics. The Scopus database showed both the highest and lowest rates, with the broader (uncorrected) search possibly overestimating the retraction rates due to false positives, and the more specific (corrected) search possibly underestimating it since only a few retracted publications were identified. In contrast, estimates for radiology were more consistent. The observed differences between these retraction rates suggest a potential gap.

At least three factors should be considered. First, radiomics is highly technical and interdisciplinary, making unintentional errors more likely. Previous studies estimated that 10–15% of radiomics publications could contain methodological errors, rendering them invalid [9, 10]. Second, the observed retraction numbers may be misleading, since many retractions occurred in general journals, although the majority of radiomics studies are published in specialized radiological or oncological journals. It remains unclear why retractions have not occurred there, despite likely similar rates of error or misconduct. Third, retraction rates are generally increasing [14]. However, after a sharp peak in 2023, the number of retracted publications in radiomics decreased. Taken together, these factors suggest that the current retraction rates are unreasonably low and likely underestimate the true extent of problematic publications.

This gap may be attributed to several reasons. Because radiomics requires specialized expertise, editors and reviewers may lack the knowledge to identify technical or methodological errors. Misconduct is equally difficult to detect from publication alone, and the current hype surrounding radiomics may lead to overly lenient editorial standards. Moreover, retractions can damage a journal's reputation, making editors hesitant to pursue them. Indeed, retractions are often borderline decisions, especially in cases of unintentional errors. For example, applying feature normalization to the entire dataset constitutes a methodological error; however, it leads to false-positive results only in rare cases [20]. Taken together, the current peer-review system is likely in part responsible for these shortcomings. Although it has been subjected to considerable criticism, there is presently no clear path forward to address these challenges [21, 22].

The low retraction rates may also have contributed to the broader ‘reproducibility crisis’, as recurring methodological errors are likely to persist and be repeated [23], although recent improvements have been observed [24]. In this sense, the situation might be viewed as a potential “retraction crisis of radiomics”.

While the direct impact on patients is limited, since it is widely recognized that even otherwise sound studies cannot be directly applied in clinical settings without thorough validation, the greater harm lies in damaging the field’s credibility. Publications containing methodological flaws that remain unretracted undermine their reputation, and may discourage researchers from remaining in the field [25]. Paradoxically, incorrect studies often receive more attention and citations [26], amplifying their influence and long-term consequences.

There is limited current research on retractions in radiology. Rosenkrantz found that retractions were uncommon but increasing, and the issue was ‘inconsistently and insufficiently addressed’ [27]. A more recent review by Qi et al showed that retractions in oncology account for nearly 20% of all retracted papers in 2021, with misconduct being the primary cause [28]. Kwee et al identified 192 retractions from the RWD database, one-third from China, with misconduct involved in over half the cases [14]. They also surveyed authors regarding fraud in their departments, concluding that scientific fraud remains a genuine concern [29]. The findings in this study are less conclusive because almost no retractions have occurred in radiomics. However, given that radiomics is a subfield of radiology, it is unlikely that radiomics is immune to similar issues. The issue of retractions extends beyond radiology and radiomics, affecting medical research more broadly. Previous studies have highlighted widespread concerns about publication bias, misconduct, and irreproducibility across biomedical fields, underscoring the need for further investigation into the prevalence and causes of retractions [30, 31].

Currently, there is no standardized or impartial mechanism to report suspected misconduct beyond directly contacting journal editors. This approach is inherently biased, as editors may be inclined to downplay or ignore such reports to prevent reputational damage. Moreover, few researchers are motivated to investigate or replicate others’ work, as such efforts bring little academic reward. Under the currently prevailing ‘publish-or-perish’ paradigm, replication studies are still undervalued, and journals are reluctant to publish negative findings.

One possible approach to improve the validity of radiomics research is the establishment of a dedicated scientific working group tasked with identifying clear unintentional and methodological errors in published radiomics studies. While initiatives such as the EuSoMII Radiomics auditing group represent an important step toward improving standards in the field and have recently published reliable checklists and guidelines [6, 32, 33], currently, their focus remains largely general, assessing overall quality standards and methodological rigor. By contrast, the proposed working group would evaluate specific publications and reproduce highly cited studies to provide authoritative assessments of reliability. Although the methods differ, the goals are aligned, to ensure methodological rigor from the outset. Methodological errors could be more reliably detected if code and data were shared, yet such practices remain uncommon. Even when shared, reviewers often lack sufficient time or expertise, potentially compromising review quality. Despite recognition of these challenges [34] and proposed reviewer guidelines [35], implementation in radiomics remains limited.

A few limitations are present in this analysis. Since radiomics evolved from texture analysis, restricting the search to “radiomics” introduced bias by excluding earlier texture analysis studies. However, texture analysis typically involves only a few features, and is therefore potentially less complex, with a lower risk of methodological errors compared to radiomics. Retractions involving exclusively deep learning methods were not investigated, and only original articles were included. Review articles, which primarily summarize findings, may be less prone to misconduct and errors. The estimated retraction rates for radiology could be biased, since the time to retraction was generally longer for these articles.

## Conclusion

In conclusion, the number of retracted publications in radiomics appears lower than in radiology, despite the presence of known methodological and reproducibility issues in the field. This may indicate that current retraction practices do not fully capture all flawed studies, although the true extent of the gap remains uncertain. The establishment of a dedicated working group to systematically assess methodological validity could be one approach to help address this potential gap.

## Abbreviations

RWD: Retraction watch database
WoS: Web of Science
